# Open-Source Image Analysis Software Yields Reproducible CT Measures of Longissimus Muscle Area and Density in Sheep

**DOI:** 10.1111/vru.70020

**Published:** 2025-03

**Authors:** Jeryl Jones, Anna Brewer, Susan Duckett, Cerano Harrison, Nataly Wickstrom, Aliute Udoka, Maslyn Greene

**Affiliations:** 1 Department of Animal and Veterinary Sciences, Clemson University, Clemson, South Carolina, USA; 2 College of Veterinary Medicine, University of Georgia, Georgia, USA; 3 South Carolina Translational Research Improving Musculoskeletal Health Center, Rhodes Research Center, Clemson University, Clemson, South Carolina, USA; 4 University of Glasgow School of Biodiversity, One Health, and Veterinary Medicine, Clemson, South Carolina, USA; 5 Life Science Facility, Clemson University Genomics and Bioinformatics Facility, Clemson, South Carolina, USA

**Keywords:** lamb, meat quality, intervertebral disc disease models, sarcopenia

## Abstract

Longissimus muscles (LM) in sheep are important for animal scientists who study meat quality and translational researchers who study thoracolumbar spinal disease. Computed tomography (CT) is an established technique for characterizing paraspinal muscles in sheep; however, studies reporting reproducibility of CT measures using open-source software are lacking. The objectives of this prospective pilot study were to develop a standardized protocol for measuring LM area and density in sheep using CT and to determine the reproducibility for measurements. Thoracolumbar CT images were acquired for four sheep at five time points each as part of another study. Six observers applied a standardized CT image analysis protocol to record triplicate transverse area (cm^2^) and water phantom-corrected mean density (Hounsfield units, HU) values for the left and right LM. Average coefficients of variation (CVs) for 4 of 6 observers were good to excellent (<10%) for all variables. Average CVs did not differ among observers for 3 of 4 variables (ANOVA, *p* > .05).

## 1 ∣ Introduction

Sheep are important sources of meat worldwide and are also commonly used as translational research models for spinal disease studies [1-4]. For meat quality researchers, the thoracolumbar portions of the longissimus muscle (LM) group in sheep are important because they are primary components of the highest value retail cuts such as “chops”, “rack of lamb”, and “loin” [5]. For translational researchers, sheep LM measures can be helpful for characterizing effects of novel treatments on experimentally induced paraspinal sarcopenia [3]. Reproducible methods for quantifying paraspinal muscle size and density can also be helpful for assessing responses to treatments in dogs and humans with naturally occurring muscle atrophy due to spinal disease [6-8]. CT is an established technique for noninvasively quantifying paraspinal muscle characteristics in sheep [9-21]; however, studies describing reproducibility of CT measures using open-source image analysis software are currently lacking. The objectives of this study were to develop and assess intraobserver reliability and inter-observer repeatability for a standardized protocol to quantify the area and density of thoracolumbar LM in sheep using CT and open-source image analysis software. Our working hypothesis was that CT measures using this protocol would be reproducible within and between observers.

## 2 ∣ Methods

### 2.1 ∣ Selection and description of subjects

The study was a prospective pilotdesign and part of a larger research study on paraspinal muscle quality in lambs. All procedures were approved by and conducted in accordance with the requirements of our university’s Institutional Animal Care and Use Committee (Clemson University IACUC, AUP 2022-0344). Four male Texel x Suffolk sheep (53 kg body weight, 7 months of age) were obtained from our university’s research and teaching sheep farm (Piedmont Research and Education Center, Clemson University) and transported to the university’s animal research center (Godley Snell Research Center, Clemson University). Sheep were housed and managed by animal research center personnel throughout the study using their standard operating procedures. CT scans of the thoracolumbar spinal region were acquired at five time points for each sheep as part of another experimental study [22]. For that study, an intramuscular injection of antagomiR-22-3p was administered into the left LM at the level of the 12th rib. An intramuscular injection of phosphate-buffered saline was administered into the right LM at the level of the 12th rib as a control. These intramuscular injections were repeated for three consecutive days. The lambs were CT scanned pretreatment and then once weekly for 4 weeks.

### 2.2 ∣ CT technical parameters

**FIGURE 1 ∣:**
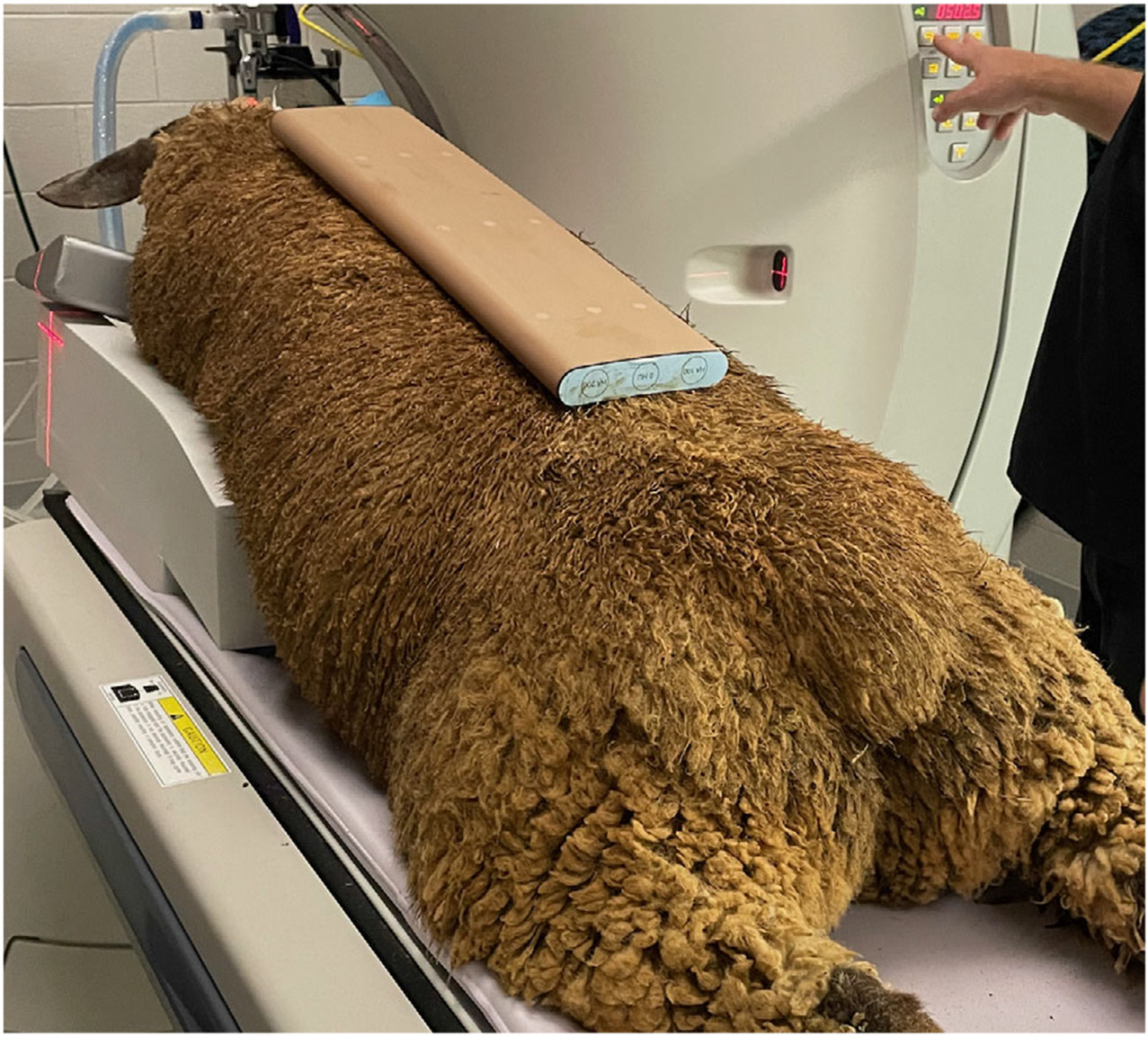
Positioning used for CT scanning of the thoracolumbar spine in an anesthetized sheep. A bone calibration phantom was placed dorsal to the thoracolumbar spine so that a water rod (known density value 0 mg/cm^3^ hydroxyapatite) would be included in the scan and used as a correction factor for LM density values.

Animals were anesthetized for CT scanning per the IACUC protocol and the research center’s attending veterinarian. Research center staff acquired scans using a 16-slice CT scanner (Toshiba Aquilion TSX-101A, GE Healthcare, Chicago IL) and a standardized protocol developed in consultation with an ACVR-certified veterinary radiologist (J. J.). Animals were positioned in sternal recumbency on a table pad with the hind limbs extended caudally, and a calibration phantom (QRM Quality Assurance in Radiology and Medicine, Möhrendorf Germany) was placed dorsal to the thoracolumbar spine (Figure 1). Scans were acquired from the caudal margin of the scapula to the cranial margin of the ilial wing (1.0 mm slice thickness, mA 400, large focal spot, thoracolumbar spine protocol, and standard algorithm). After the final CT scan for each sheep, a line was drawn on the surface of the skin with a permanent marker to identify the location of the T12-13 CT transverse slice so researchers could match this with the transverse muscle section used for subsequent tissue analyses.

### 2.3 ∣ CT Image Analysis

**FIGURE 2 ∣:**
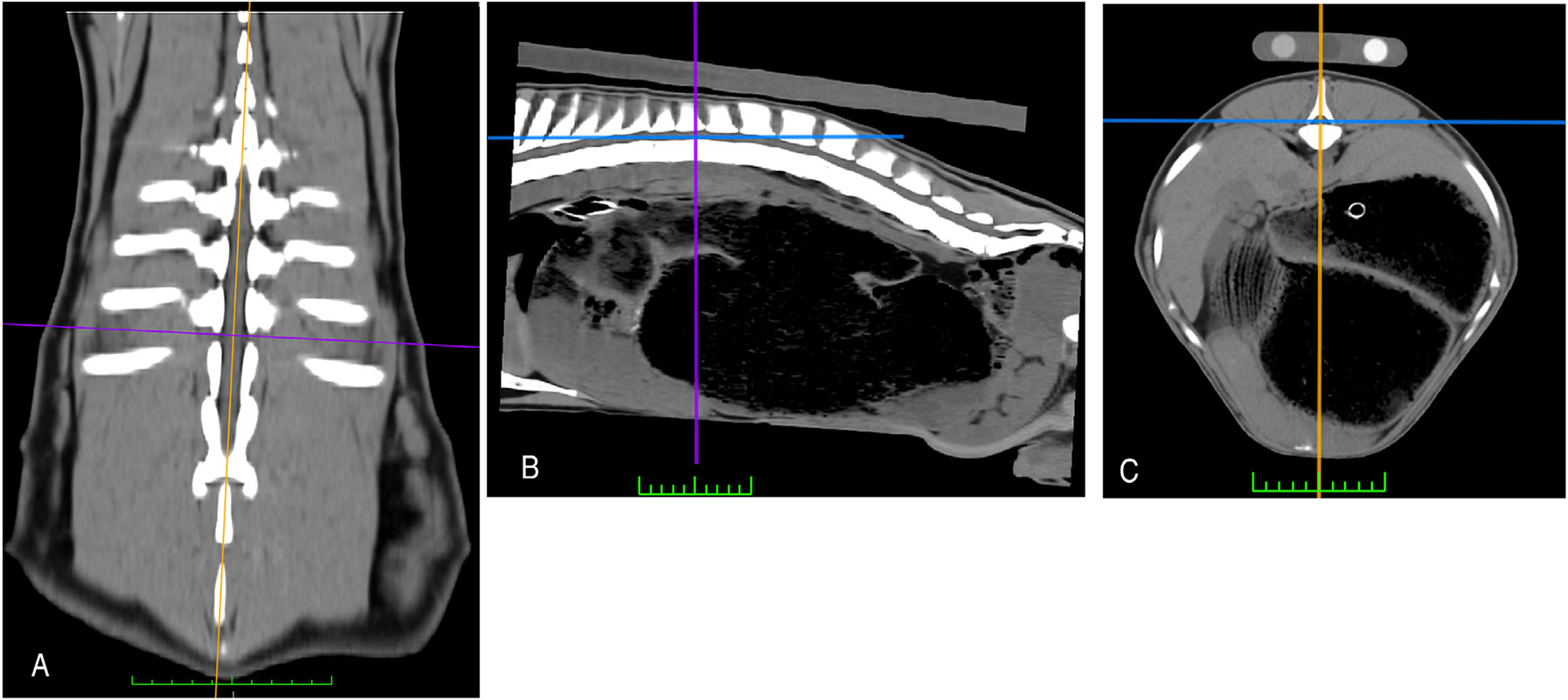
Soft tissue window, 3D MPR CT images illustrating how slice orientation and location decisions were made for transverse slices used in muscle measurements. A, Dorsal planar. B, Sagittal planar. C, Transverse planar (full protocol in Supporting Information S1).

**FIGURE 3 ∣:**
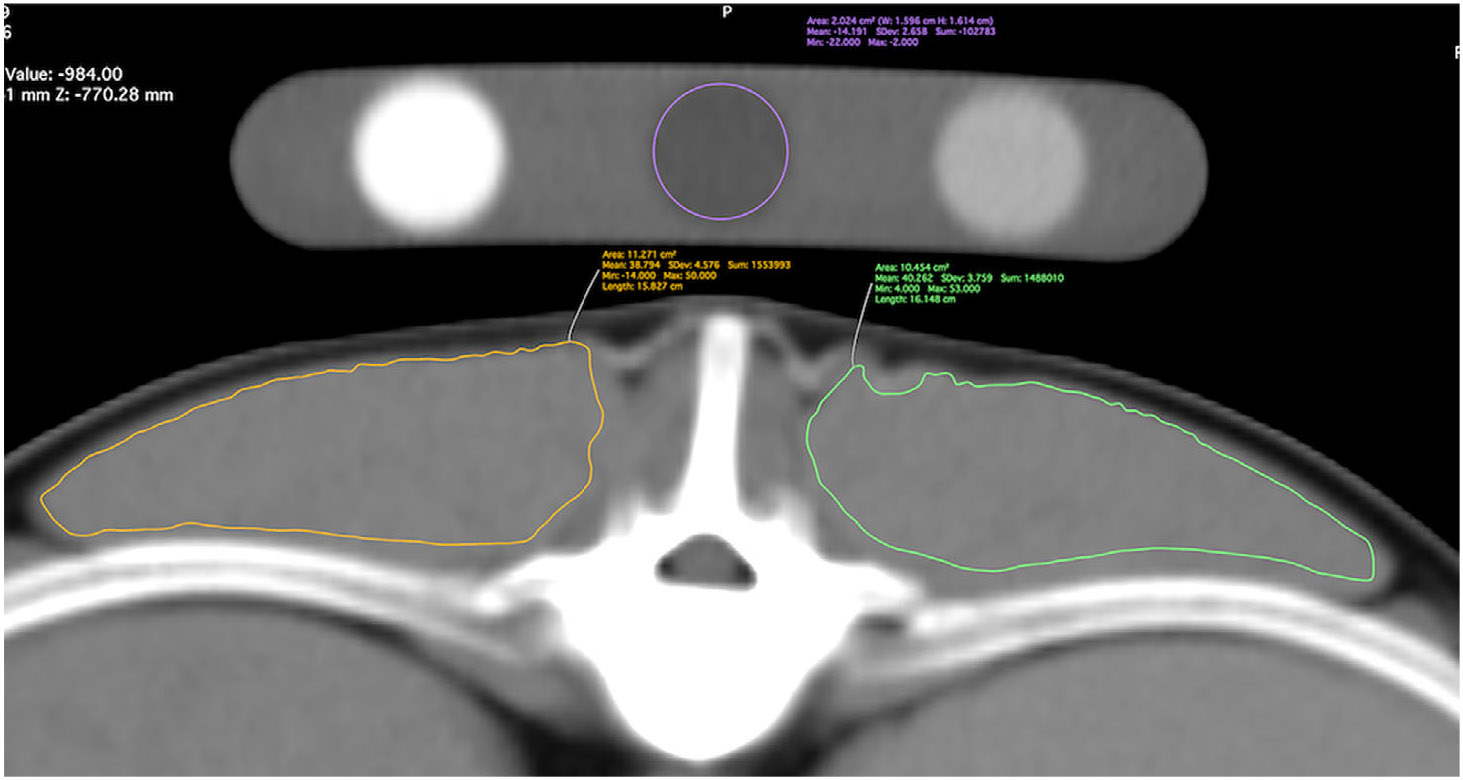
Soft tissue window, 3D MPR thick slab, transverse CT image illustrating how regions of interest were created for measuring right and left LM area and mean density, and mean density of the water calibration rod in the phantom.

Image analyses were performed using an image analysis workstation and open-source image analysis software (Mac OS High Sierra, 10.13.6, MacPro Quad Core, Apple, Inc. Cupertino, Ca; Horos https://horosproject.org/). A standardized CT image analysis protocol was designed by two undergraduate research students in consultation with research project team members (JJ, AB, SD, CH, AU, MG) (adapted from Keller et al. [8] and Cain et al. [6]). A detailed, step-by-step protocol description is provided in Supporting Information S1. One of the undergraduate student protocol designers (N. W.) then trained an MS student (A. B.) and four additional undergraduate research students to apply the protocol for intraobserver reliability and inter-observer repeatability studies. These six observers independently performed triplicate measurements of transverse sectional area (cm^2^) and mean density (Hounsfield units, HU) for right and left longissimus muscles (LM) at T12-13 (Figures 2 and 3). The mean density of the calibration phantom’s water rod at the T12-13 location was also recorded. The order of scans and the right/left order for LM were randomized for each measurement session. Readers were aware of the treatments that were administered but unaware of the scan time point at the time of interpretation.

### 2.4 ∣ Statistics

Statistical analyses were selected and performed by the MS student (A. B.) in consultation with a PhD student (C. H.) and a statistician. All analyses were performed using commercial software (JMP PRO 17, SAS Institute Inc.; SAS 9.4, SAS Inst. Inc., Cary, NC, USA). A power analysis was not performed. Corrected mean CT density values were calculated using the following formula: mean LM CT density (HU) – mean water calibration phantom CT density (HU). For each of the four variables of interest (left area, right area, left corrected density, and right corrected density), the coefficient of variation (CV) of triplicate measurements was calculated using the following formula: CV = [standard deviation/mean] × 100. For intraobserver reliability analyses, the CVs for each observer were first averaged across weeks (for each animal) and then averaged across animals. For interobserver repeatability analyses, average CVs were compared across observers using an analysis of variance (ANOVA) followed by pairwise Student’s *t*-tests to determine specific differences in average CVs between the observers. *p*-values (denoted *p*) less than .05 were considered evidence of significant differences in average CVs between observers.

## 3 ∣ Results

A total of 18 CT scans were included in the reproducibility analyses. One of the sheep did not recover from anesthesia after the third CT scan. The remaining three sheep recovered normally and had no postanesthesia complications.

For LM area intraobserver reliability analyses, average CV values for Observer 1, 2, 3, 5, and 6 were <10%, and values for Observer 4 were <13% (Supporting Information S2). For interobserver repeatability analyses, average CV values did not differ among observers for the left side (F-ratio = 1.6969, *p* = .20) but differed among observers for the right side (F-ratio = 8.5, *p* < .0004) based on ANOVA. Pairwise Student’s *t*-tests were next performed to see which observers had average CVs that differed (Supporting Information S3). The average CV for observer 4 was significantly different from those of observers 1, 5, and 6 (*p* < .05).

For LM corrected CT density intraobserver reliability analyses, average CV values for observers 2, 3, 5, and 6 were <10%, and values for observers 1 and 4 were <16% (Supporting Information S2). For inter-observer repeatability analyses, average CV values did not differ among observers but were close to the threshold based on ANOVA (left, *p* = .051; right, *p* = .064). Pairwise Student’s *t*-tests detected differences between some observers (Supporting Information S3). The average CVs for observer 4 differed from observers 1, 5, 6 for the left area; observers 1 and 4 differed from observers 2 and 6 for left density; observer 4 differed from observers 1, 2, 3, 5, 6 for the right area; and observers 1 and 4 differed from observers 2 and 6 for right density (*p* < .05).

## 4 ∣ Discussion

The primary intentions of the current pilot study were to support long-term goals for this research team and future longitudinal studies for other researchers evaluating paraspinal muscle quality in sheep. The study evaluated the precision of LM CT measurements using a standardized image acquisition protocol, a standardized image analysis protocol, 18 CT scans acquired at varying time points, and 6 observers with varying levels of experience. Our working hypothesis was supported in that average CVs for 4 of 6 observers were <10% for all variables. Average CVs did not differ among observers for 3 of 4 variables (*p* > .05). Student’s *t*-test analyses indicated that measurements for two observers were outliers, but measurements for the other four observers were reproducible. The authors retrospectively debriefed one of the observers with measurement outliers and proposed the following reasons: insufficient understanding of the normal LM anatomy and eye strain, which made visualization of borders more difficult. A previous study found that reminders to take breaks every 20 min during computer use reduced some measures of digital eye strain and dry eye in a sample of healthy volunteers [23].

One previous study described the measurement of LM width and depth in sheep using CT and the repeatability of these measurements [9]. The authors used proprietary software, and their measurements were made at the 5th lumbar vertebra. Based on intraclass correlation analyses, authors reported intraobserver reliability percentages ranging from 95–99% and inter-observer repeatability percentages ranging from 90 to 92%. Three publications described CT image analysis techniques for predicting intramuscular fat (fat %) [11, 14, 21]. We did not measure LM fat % in our study because our open-source software program did not have a plug-in for making this measurement. Instead, we measured corrected mean CT density values as indirect measures of intramuscular fat, based on the assumption that increased intramuscular fat would cause a decrease in muscle density. For each corrected mean CT density value, we subtracted the mean CT density of the calibration phantom’s water rod from the mean CT density of the muscle to help minimize outside sources of variation. We used the “thick slab” tool in our 3D MPR software to match the thickness of the CT slice with the thickness of the muscle sample researchers planned to use for their other tissue analyses. We used the CT laser light and a line drawn with a permanent marker on the surface of the skin to help researchers match the location of the T12-13 CT slice in the final scan with the location of their muscle sample. We positioned sheep in sternal recumbency for each CT scanning session to match positioning that would be used for muscle injections and postmortem muscle dissections. This positioning was also intended to help minimize outside sources of variation on CT measures of muscle size due to compression forces that would be expected with dorsal or lateral recumbency.

In addition to applications for researchers studying meat quality in sheep, the methodologies introduced in the current study could be useful for a wide variety of other applications and species. Examples may include monitoring changes in paraspinal muscle size and density due to injury, disease, physical therapy, surgery, and/or other treatments. When determining whether CT imaging is appropriate for longitudinal studies, authors recommend that researchers assess the risks versus benefits for repeated anesthetic sessions. Sheep can be especially sensitive to anesthesia [24]. In the current longitudinal study, no anesthesia complications occurred for weekly CT scanning sessions in three of the sheep. However, one of the sheep did not recover from anesthesia after the third scanning session.

Limitations of the current study were small sample sizes and a lack of CT measurement accuracy analyses. To complete other time-sensitive tissue analyses at the end of the study, researchers decided to excise the LM from surrounding skin, bone, and connective tissue attachments [22]. Without surrounding supportive structures, the shape of the excised LM changed and precluded accuracy analyses for CT measures of transverse area versus actual measures of transverse area.

In conclusion, the current study introduced a reproducible, standardized protocol for quantifying CT area and corrected density for the thoracolumbar portion of the LM group in sheep using open-source image analysis software. If accuracy analyses for CT area measurements are needed for future longitudinal studies, the authors recommend keeping muscles attached to surrounding structures for transverse slicing and obtaining high-resolution photographs of the slices before excising the muscles. Additional observer training on normal LM anatomy and reminders to implement regular breaks during data recording sessions are also recommended. Future studies using larger sample sizes and a longer period would be warranted to determine the diagnostic sensitivity of this protocol for discriminating untreated versus treated muscles. Future studies are also needed to evaluate this protocol as a potential tool for monitoring changes in paraspinal muscles for other species with experimentally induced or naturally occurring muscle disease.

## Supplementary Material

Supplement 2

Supplement 3

Supplement 1

## Data Availability

Data are available from the corresponding author upon reasonable request.
