# Supplementary material for: Open-Source Image Analysis Software Yields Reproducible CT Measures of Longissimus Muscle Area and Density in Sheep
Source: Vet Radiol Ultrasound. Author manuscript; Available in PMC 2025 Apr 16. (PMC12000905; doi:10.1111/vru.70020)
Supplement: Supplement 2 [file NIHMS2072323-supplement-Supplement_2.pdf]

Supplement 2. Results of intra-observer reliability analyses for longissimus muscle CT measurements of area and corrected density at T12-13 in sheep.

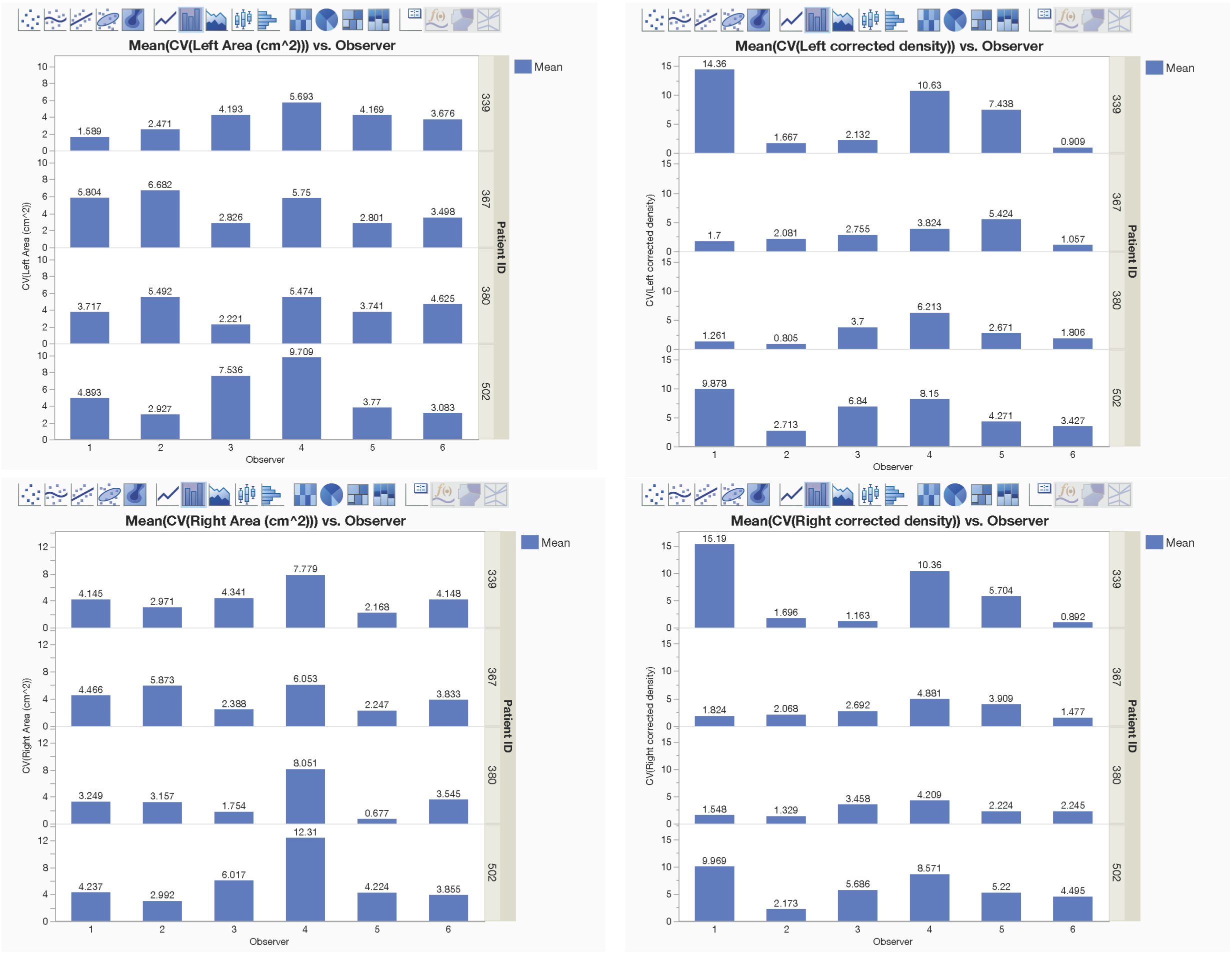

Notes: CV, coefficient of variation; corrected density = (mean HU muscle – mean HU water phantom); LM, longissimus muscle; Std, standard. Average CVs were <10% for Observers 2, 3, 4, 5. Observers 1 and 4 had average CVs between 10-16%.
