# Supplementary material for: Open-Source Image Analysis Software Yields Reproducible CT Measures of Longissimus Muscle Area and Density in Sheep
Source: Vet Radiol Ultrasound. Author manuscript; Available in PMC 2025 Apr 16. (PMC12000905; doi:10.1111/vru.70020)
Supplement: Supplement 3 [file NIHMS2072323-supplement-Supplement_3.pdf]

Supplement 3. Results of inter-observer pairwise Student's T-test comparisons for average coefficients of variation of triplicate CT measurements of longissimus muscle area and corrected density in sheep.

Left Area

Level	- Level	Difference	Std Err Dif	Lower CL	Upper CL	p-Value
4	5	3.036200	1.224201	0.42688	5.645523	0.0255*
4	6	2.935905	1.224201	0.32658	5.545228	0.0299*
4	1	2.656017	1.224201	0.04669	5.265340	0.0465*
4	3	2.462608	1.224201	-0.14671	5.071930	0.0626
4	2	2.263641	1.224201	-0.34568	4.872964	0.0843
2	5	0.772559	1.224201	-1.83676	3.381881	0.5375
2	6	0.672264	1.224201	-1.93706	3.281587	0.5910
3	5	0.573593	1.224201	-2.03573	3.182915	0.6461
3	6	0.473298	1.224201	-2.13602	3.082620	0.7045
2	1	0.392376	1.224201	-2.21695	3.001698	0.7530
1	5	0.380183	1.224201	-2.22914	2.989506	0.7604
1	6	0.279888	1.224201	-2.32943	2.889211	0.8222
2	3	0.198966	1.224201	-2.41036	2.808289	0.8731
3	1	0.193409	1.224201	-2.41591	2.802732	0.8766
6	5	0.100295	1.224201	-2.50903	2.709617	0.9358

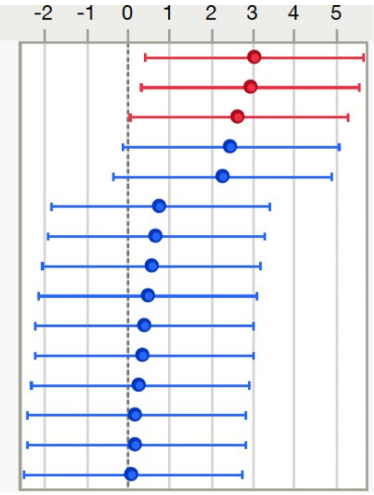

Left Corrected Density

Level	- Level	Difference	Std Err Dif	Lower CL	Upper CL	p-Value
4	6	5.405815	1.961342	1.22531	9.586317	0.0147*
4	2	5.389103	1.961342	1.20860	9.569605	0.0150*
1	6	4.999901	1.961342	0.81940	9.180403	0.0222*
1	2	4.983189	1.961342	0.80269	9.163691	0.0226*
4	3	3.348661	1.961342	-0.83184	7.529163	0.1084
5	6	3.151393	1.961342	-1.02911	7.331895	0.1290
5	2	3.134681	1.961342	-1.04582	7.315183	0.1308
1	3	2.942747	1.961342	-1.23775	7.123249	0.1543
4	5	2.254422	1.961342	-1.92608	6.434924	0.2684
3	6	2.057154	1.961342	-2.12335	6.237656	0.3109
3	2	2.040442	1.961342	-2.14006	6.220944	0.3147
1	5	1.848508	1.961342	-2.33199	6.029010	0.3609
5	3	1.094239	1.961342	-3.08626	5.274741	0.5851
4	1	0.405914	1.961342	-3.77459	4.586416	0.8388
2	6	0.016712	1.961342	-4.16379	4.197214	0.9933

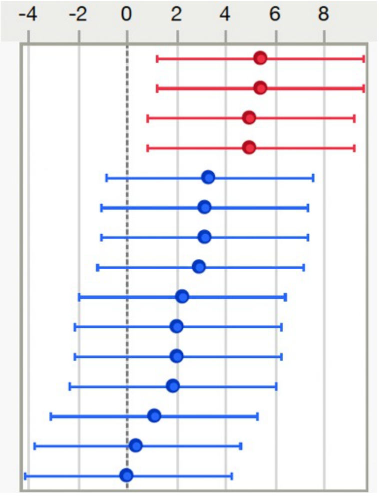

Right Area

Level	- Level	Difference	Std Err Dif	Lower CL	Upper CL	p-Value
4	5	6.219070	1.012995	4.05992	8.378217	<.0001*
4	3	4.923386	1.012995	2.76424	7.082533	0.0002*
4	2	4.799605	1.012995	2.64046	6.958752	0.0003*
4	6	4.702548	1.012995	2.54340	6.861695	0.0003*
4	1	4.523736	1.012995	2.36459	6.682883	0.0005*
1	5	1.695333	1.012995	-0.46381	3.854481	0.1149
6	5	1.516522	1.012995	-0.64263	3.675669	0.1551
2	5	1.419464	1.012995	-0.73968	3.578612	0.1815
3	5	1.295684	1.012995	-0.86346	3.454831	0.2203
1	3	0.399650	1.012995	-1.75950	2.558797	0.6987
1	2	0.275869	1.012995	-1.88328	2.435016	0.7891
6	3	0.220838	1.012995	-1.93831	2.379986	0.8304
1	6	0.178811	1.012995	-1.98034	2.337959	0.8622
2	3	0.123781	1.012995	-2.03537	2.282928	0.9044
6	2	0.097058	1.012995	-2.06209	2.256205	0.9249

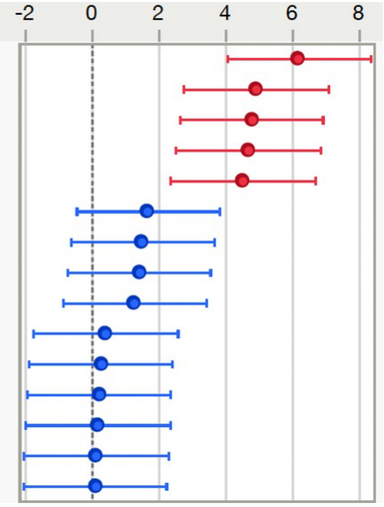

Right Corrected Density

Level	- Level	Difference	Std Err Dif	Lower CL	Upper CL	p-Value
1	2	5.316646	1.996856	1.06045	9.572843	0.0177*
4	2	5.187813	1.996856	0.93161	9.444011	0.0202*
1	6	4.856127	1.996856	0.59993	9.112325	0.0280*
4	6	4.727294	1.996856	0.47110	8.983492	0.0318*
1	3	3.883641	1.996856	-0.37256	8.139839	0.0708
4	3	3.754809	1.996856	-0.50139	8.011006	0.0796
1	5	2.868997	1.996856	-1.38720	7.125195	0.1713
4	5	2.740164	1.996856	-1.51603	6.996362	0.1902
5	2	2.447649	1.996856	-1.80855	6.703847	0.2392
5	6	1.987130	1.996856	-2.26907	6.243328	0.3355
3	2	1.433004	1.996856	-2.82319	5.689202	0.4840
5	3	1.014645	1.996856	-3.24155	5.270842	0.6188
3	6	0.972485	1.996856	-3.28371	5.228683	0.6333
6	2	0.460519	1.996856	-3.79568	4.716717	0.8207
1	4	0.128833	1.996856	-4.12736	4.385031	0.9494

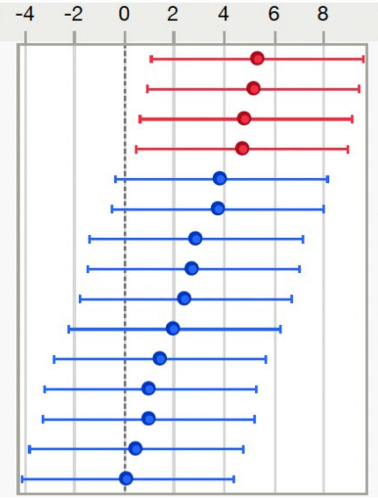

Notes: Level = observer number; Std, standard; Err, error; Dif, difference; CL, confidence limit. Comparisons between most observers did not differ. Observers 1 and 4 had values that differed from others and were interpreted as outliers.
