# Supplementary material for: Open-Source Image Analysis Software Yields Reproducible CT Measures of Longissimus Muscle Area and Density in Sheep
Source: Vet Radiol Ultrasound. Author manuscript; Available in PMC 2025 Apr 16. (PMC12000905; doi:10.1111/vru.70020)
Supplement: Supplement 1 [file NIHMS2072323-supplement-Supplement_1.pdf]

## STANDARDIZED PROTOCOL FOR QUANTIFYING THE LONGISSIMUS DORSI MUSCLE IN SHEEP MODELS USING CT MEASURES AND OPEN-SOURCE IMAGE ANALYSIS SOFTWARE

1. Launch Horos image analysis software program, by clicking on the icon on your screen that matches the one below. (www.horosproject.org)

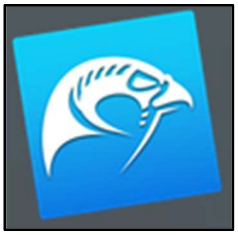

2. After Horos has successfully launched, in the far-left frame look for desired study in the albums list and click on it.

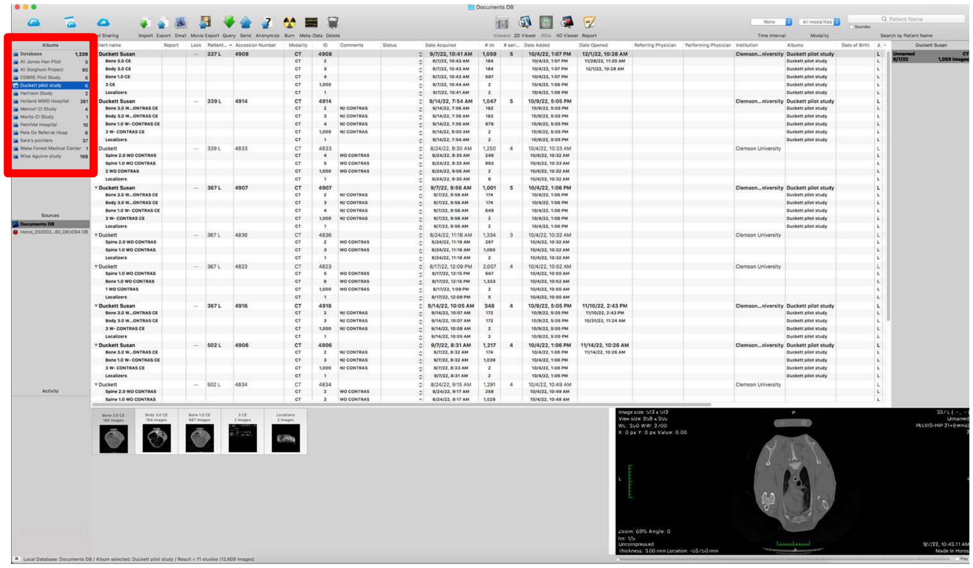

3. Find the column labeled “Accession number” four columns over to the right of the “Patient Name” column tab. This is a unique identification number for the study. Organize this column by clicking the accession number column heading to arrange them from lowest to highest.

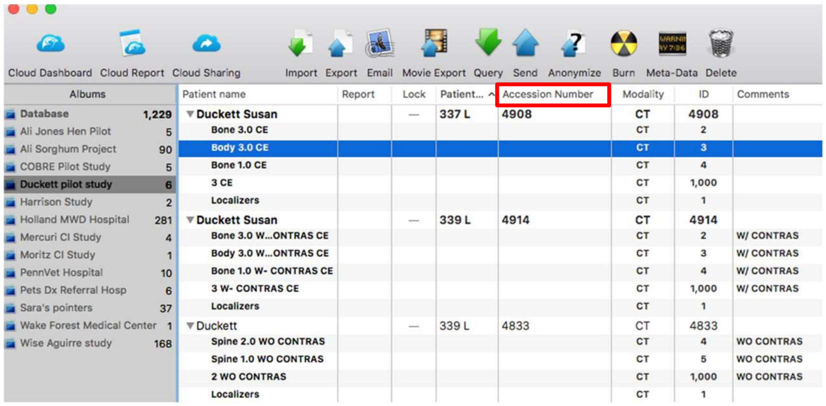


4. Assign each accession number a research number (ex. 1-12 if there are a total of 12 scans to be interpreted).
5. Randomize the order of scans using a random number generator (Random.org)
6. At the top of the screen hover over the “Numbers” header and select “Sequences” from the drop-down menu.

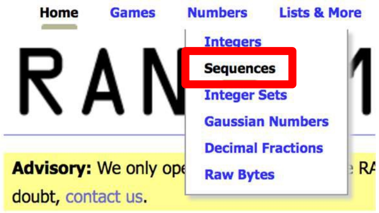


7. Now input the lowest through highest (1-18 for our purposes) and have it formatted into one column.
8. Now click on “Get sequence”, make sure to write down the corresponding accession numbers in your data table.

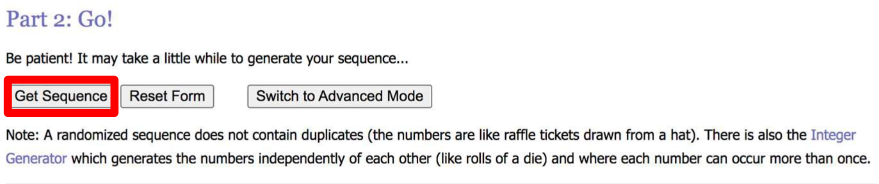

9. To repeat this for the next two replicates click on “Again!” at the bottom until you have the order of your accession numbers for each replicate written down in their corresponding data tables.
10. Write down the corresponding patient ID at this time.
11. Select the scan for the first accession number in your list by clicking the down pointing arrow next to the patient name on the scan you wish to study, then double click the body 3.0 protocol.
12. Select the “Body 3.0” scan from the list.

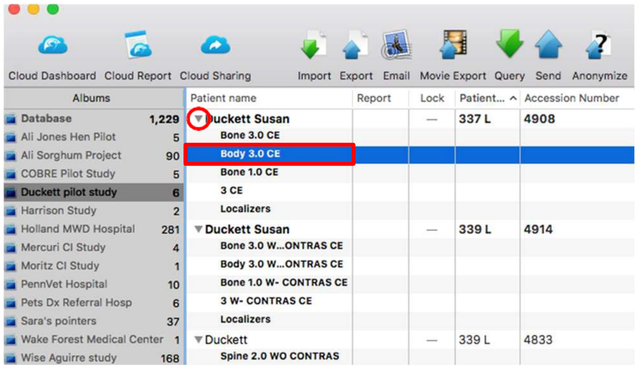


13. Once opened, set the mouse functions for the left/right button and the roller to the following: (keep in mind this may be a default)
14. Left: Density adjustment
15. Right: Zoom
16. Roller: Reposition image
17.  Note: To utilize each of these functions, the button must be depressed before moving the mouse to make the adjustment.

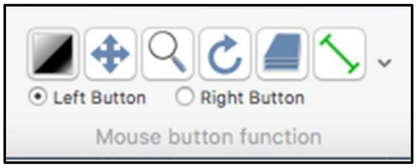

18. Set the WL/WW found to the right of the mouse functions, to the preset 2-CT- Abdomen, using the drop-down menu by clicking on the blue arrow next to the WL/WW box.

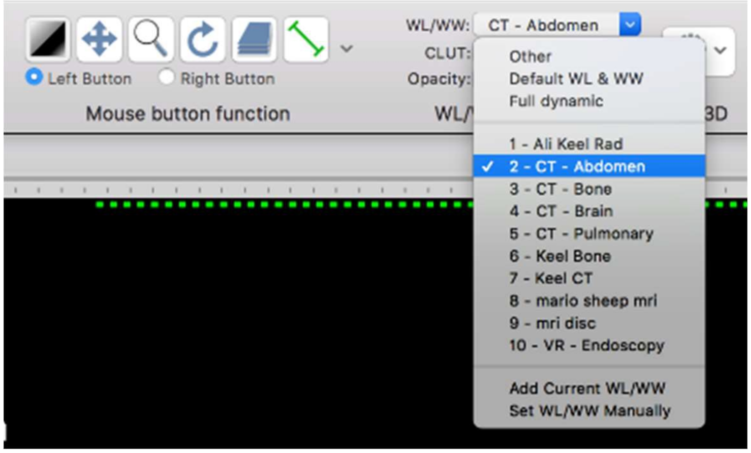


19. To set up the correct location for measurements:
20. Click on 3D viewer at the top of the screen and from the drop-down menu select 3D MPR.

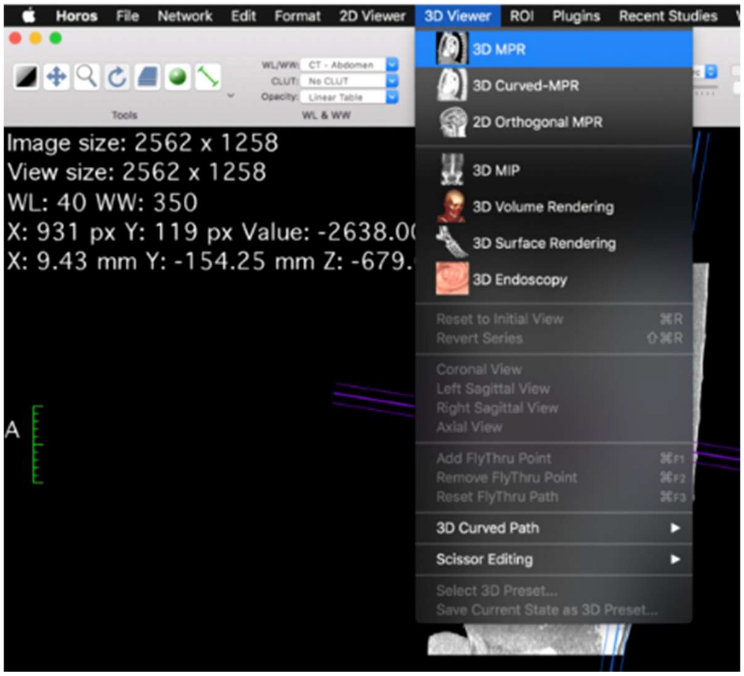

21. In the right window (dorsal planar view), use the mouse roller and scroll until you can clearly visualize the vertebral column and intervertebral discs. You want to be able to view where the spine and ribs connect. See below for an example.

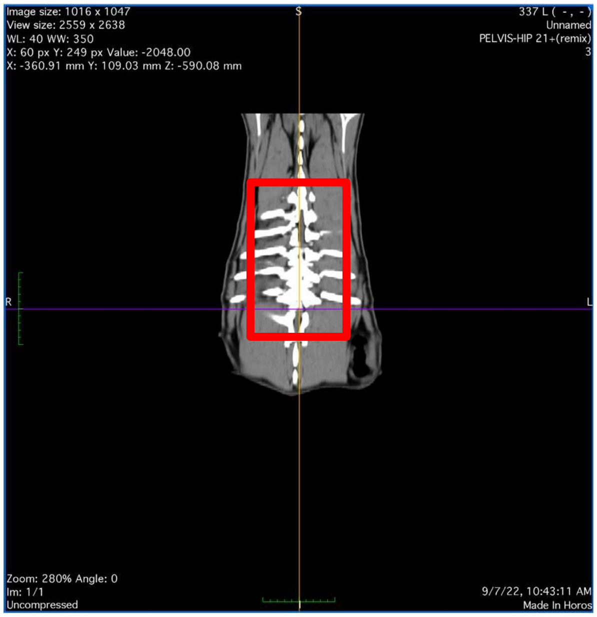


22. Click and shift the purple line so that it is centered in the space between the last two ribs (T12-13) on the vertebral column. Do this by clicking on the intersection of the two lines and dragging the line to the last two ribs.
23. Note: if you accidently rotate instead of shifting the purple line you need to select the intersection of the two lines and wait till you see the hand icon to shift the line.

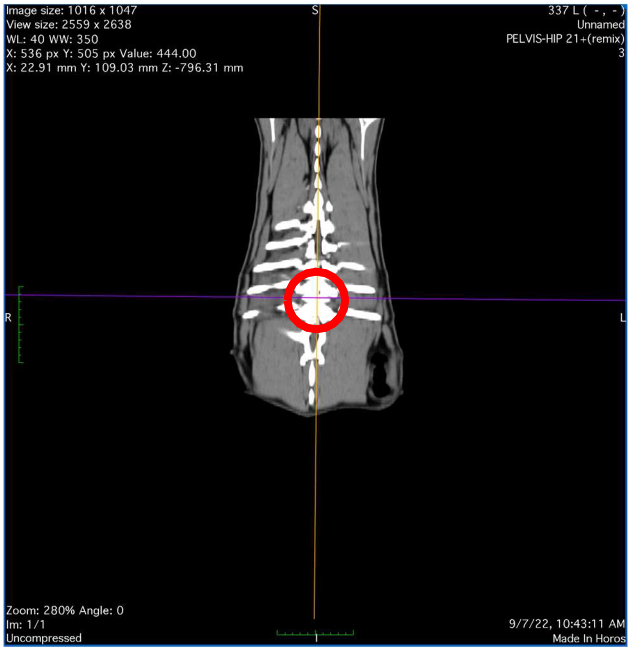

24. Click and rotate the orange line so that is parallel to the spine by clicking outside the intersection of the two lines. Make sure the purple line is also perpendicular to the spine.
25. If the right and left ribs do not appear the same in the dorsal planar view, this could be due to obliquity of patient positioning. To correct this, look in the bottom left window (transverse view) and adjust the blue line of cut. Do this by clicking on the blue line and rotating it as needed until the right and left sides of the vertebral canal are divided equally. This should make the last pairs of ribs look the same in the dorsal planar view.

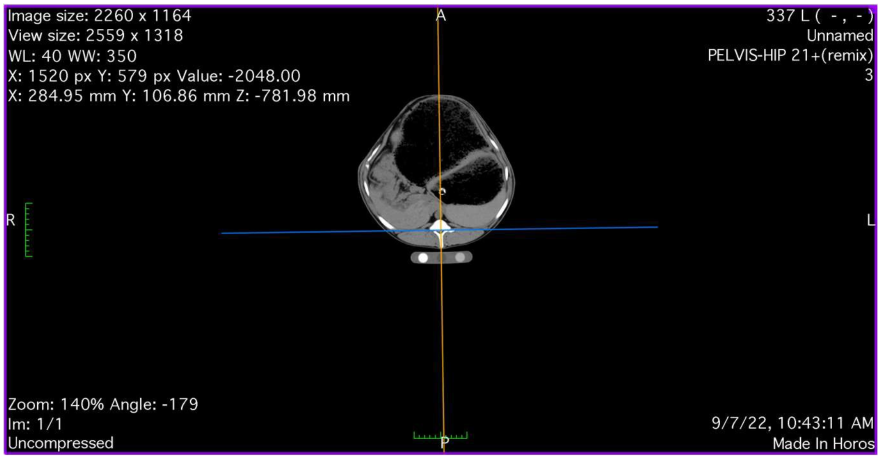


26. Find Mode in the top tool bar above Thick Slab and select the drop-down menu by clicking on the blue arrows on the end and from the drop-down menu select "mean".

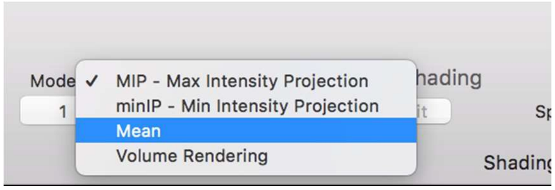


27. Set the slice thickness to 25.4mm by clicking on the white box under Mode and typing in "25.4". This adjusts the CT slice thickness to match the tissue sample thickness.
28. Recheck the dorsal planar view and make sure your slice is still centered between the last two ribs. If not adjust as needed by repeating the previous steps b - e as needed until line placement is correct.

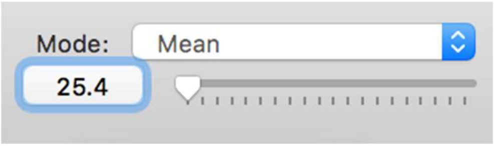

29. Now with the slice set we need to adjust the longissiumus muscle to make it best for measuring:
30. Double click on the transverse view to enlarge it.
31. To flip the image so that the spine is at the top you will click on 2D viewer go down and hover over orientation till the options pull out to the right and select Flip Image Vertical.

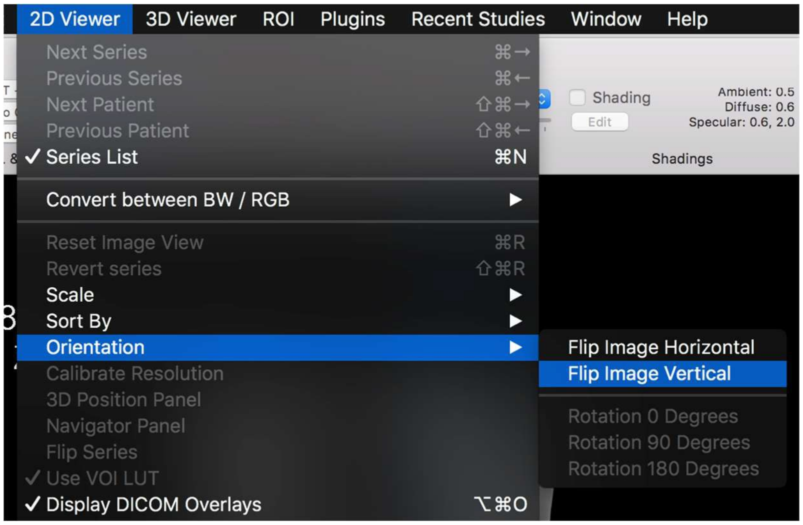


32. Zoom in on the portion we will be measuring by taking your mouse and using the right button to zoom in and by pressing on the roller as a button to pan the image around on the screen. It should look like below.
33. Note: If you accidently adjust the WW/WL settings go to the top and select 2-CT-Abdomen again (step 11). If you are unsure if you changed them, repeat step 11 as a precaution.

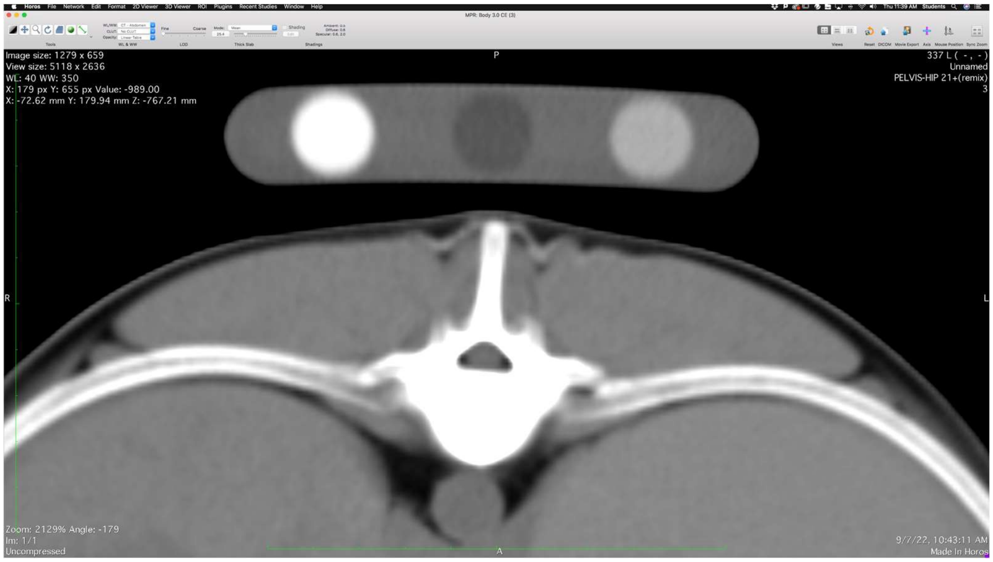

34. To measure the water phantom:
35. Select the oval tool by clicking on the arrow on the right of tools and select oval from the drop-down menu

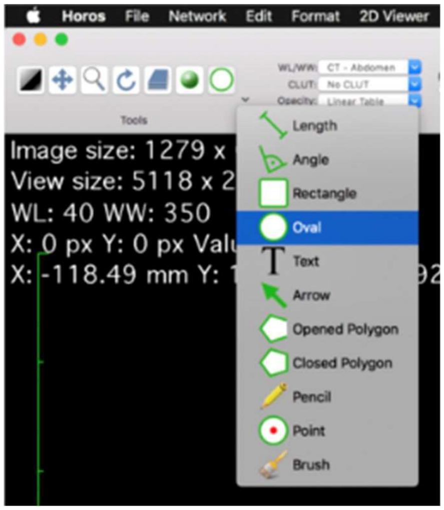


36. Center your cursor over the water phantom, the middle circle in the beam over our patient image and hold down the left mouse button and drag out to create a circle covering only the water phantom. It is important that only the water phantom is included and nothing else, the water phantom will act as a way of balancing the scale when measuring the mean density for the muscles.
37. Once you've created the circle release your button, once you've released you should see the measurements pop up in a window. The software will produce the area measurement, mean CT density, and standard deviation of CT density. Record these values in your data recording sheet.
38. Note: you can move these a round so they do not get in your way by clicking on them and dragging them to where you want them

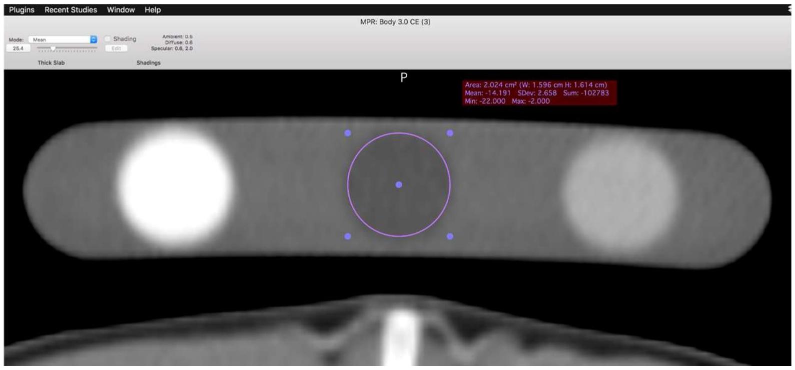

39. Assign what side you will start measuring the scan, right or left muscle, by flipping a coin. The heads facing up will mean you start by measuring the left side first and tails facing up will mean you start measuring the right side first. Flip a coin each time you open a scan. Write down what you flipped, heads or tails, next to the “Accession Number” column in the data tables. (ex. if you have 12 total scans and you need to make measurements 3 times you will flip a coin 36 times total)
40. To measure the muscle:
41. Select the pencil tool by clicking on the arrow on the right of tools and select pencil from the drop-down menu
42. Hold down the left mouse button to trace the outer margins of the longissiumus muscle. Repeat this process for the opposite side you did not already measure.

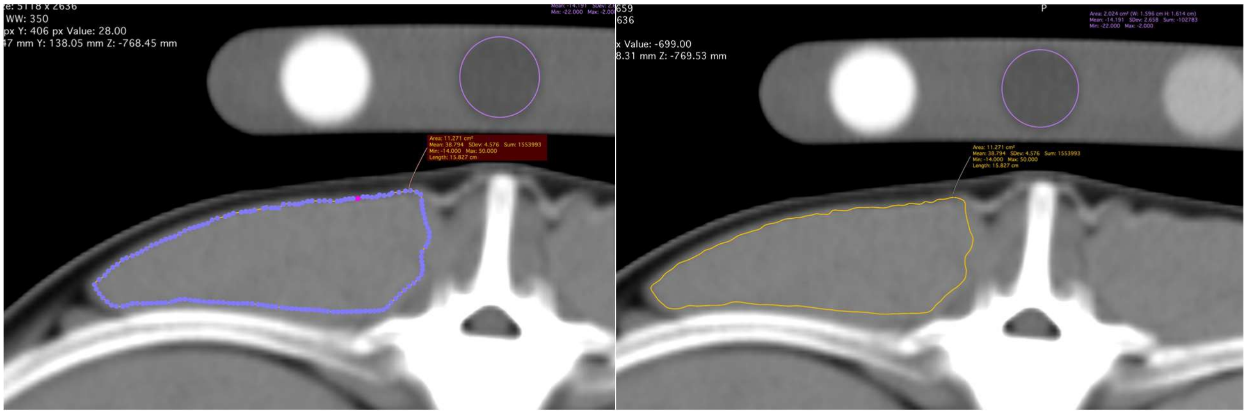


43. Record the traced area of the muscle, CT density (mean), and standard deviation within the data recording sheet table for each side.

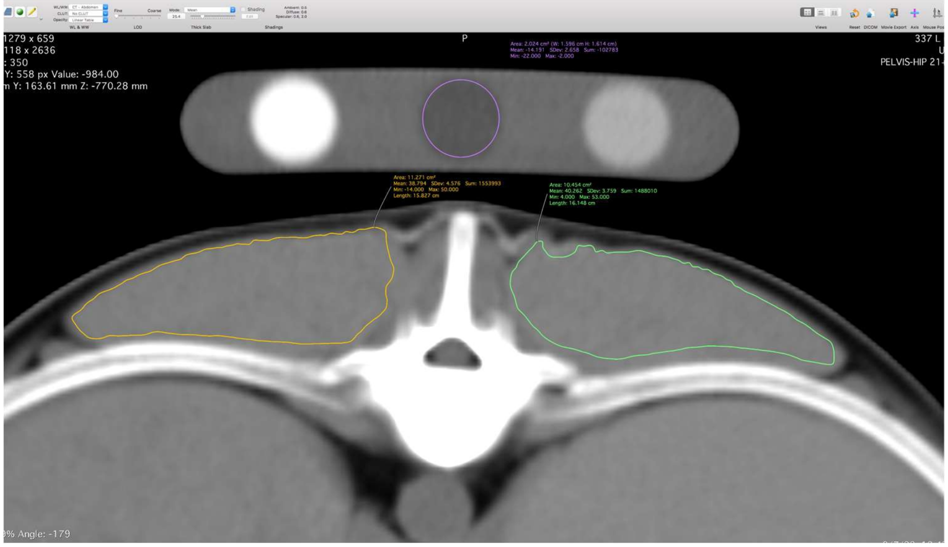

44. Delete all your measurements by clicking on “ROI” at the top and selecting Delete All ROIs in this Series

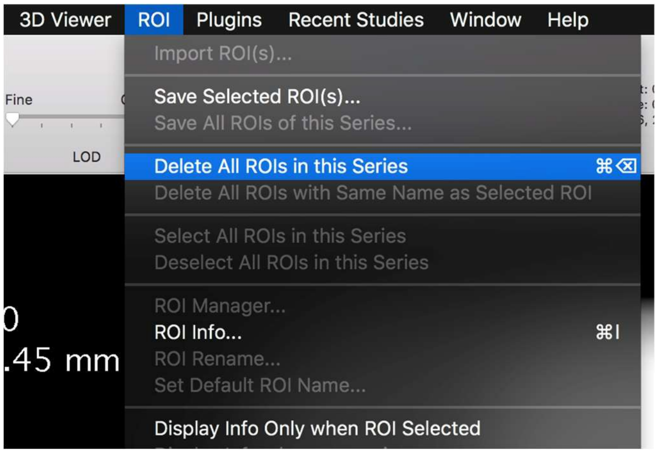


45. Repeat the above steps for the next accession number in your randomized list.
46. When values for all accession numbers have been entered into your data sheet, put the data sheet in the notebook and get a new blank data sheet.
47. Record your name, the new date(s) you made your measurements, and the new replicate number at the top.
48. Re-randomize the list of accession numbers and enter these into your new data sheet.
49. Repeat these steps until you have completed three replicates for all the accession numbers.
50. Notify PI when you have finished making all your measurements so she can enter your findings into the data spreadsheet she will be using for her analyses.
